# Accuracy of the ^14^C-urea breath test for the diagnosis of *Helicobacter pylori*

**DOI:** 10.1590/S1516-31802002000300002

**Published:** 2002-05-02

**Authors:** Ana Thereza Britto Gomes, Luciano Kowalsky Coelho, Marie Secaf, José Luiz Pimenta Módena, Luiz Ernesto de Almeida Troncon, Ricardo Brandt de Oliveira

**Keywords:** *Helicobacter pylori*, ^14^C-urea breath test, Peptic ulcer, *Helicobacter pylori*, Teste respiratório, Úlcera péptica

## Abstract

**CONTEXT:**

The development of simple, accurate and low-expense techniques for detection of *Helicobacter pylori* infection has great relevance.

**OBJECTIVE:**

To determine the accuracy of a rapid ^14^C-urea breath test (UBT) employing a very simple device for breathed air collection.

**DESIGN:**

Prospective study.

**SETTING:**

Hospital das Clinicas of the Faculty of Medicine of Ribeirão Preto.

**PARTICIPANTS:**

One hundred and thirty-seven adult patients who underwent upper gastrointestinal endoscopy in the Clinical Hospital.

**MAIN MEASUREMENTS:**

Histology for *Helicobacter pylori* (HP); urease test; urea breath test (UBT).

**RESULTS:**

One hundred and fifteen patients were infected by HP (HP+) according to both histology and the urease test, and 22 patients were HP-negative (HP-), according to the same two tests. UBT was capable of discriminating between HP+ and HP- in a way that was similar to the combination of urease test and histology. When this combination of results is taken as the "gold standard" for HP infection, the sensitivity and specificity of UBT are both greater than 90% for a range of cut-off points and breathed air collection times.

**CONCLUSION:**

The rapid UBT employing a simple device for air collection has a high accuracy in determining HP infection.

## RESUMO

**CONTEXTO:**

O desenvolvimento de técnicas simples, acuradas e de baixo custo para a detecção da infecção do *Helicobacter pylori* tem grande interesse.

**OBJETIVO:**

Determinar a acurácia de um teste respiratório rápido (UBT) que emprega dispositivo simples para coleção do ar expirado.

**TIPO DE ESTUDO:**

Estudo prospectivo.

**LOCAL:**

Hospital das Clínicas da Faculdade de Medicina de Ribeirão Preto.

**PARTICIPANTES:**

137 pacientes adultos que realizaram endoscopia digestiva alta no HCFMRP e consentiram em participar da pesquisa.

**VARIÁVEIS ESTUDADAS:**

Histologia e teste da urease, UBT. Variáveis estudadas: sensibilidade e especificidade do UBT.

**RESULTADOS:**

115 pacientes estavam infectados pelo HP conforme os resultados tanto da histologia como do teste da urease e 22 eram negativos para HP de acordo com os mesmos testes. O UBT discriminou claramente esses dois grupos, e quando a combinação de histologia e teste da urease foi tomada como "gold standard" para infecção pelo HP, a especificidade e a sensibilidade do UBT foram superiores a 90% para diferentes pontos de corte e momentos de coleta do ar expirado.

**CONCLUSÃO:**

UBT rápido tem elevada acurácia para a infecção pelo HP.

## INTRODUCTION

The discovery of the association between *Helicobacter pylori* (HP) and peptic ulcer disease by Warren and Marshall more than a decade ago^1^ was a remarkable breakthrough in the understanding of upper gastrointestinal (GI) disease. Since then, the question of how to detect this bacterium in clinical routine has gained enormous importance. Currently, there are several accurate methods for HP detection, even though none is perfect.

The ideal approach for primary diagnosis of HP is to perform an endoscopy to obtain biopsy specimens for histology, the urease test or culture, or both, whereas the urea breath test (UBT) is considered the best choice for the diagnosis of HP infection after treatment when it is not necessary to perform endoscopic reevaluation on a patient, such as in case of duodenal ulcer.^2-3^

The UBT is simple, robust, noninvasive, accurate and inexpensive.^4^ In our institution, each test costs 70 reais excluding the price of the beta counter. Its rationale is the large amount of urease produced by HP: when urea labeled with a carbon isotope is ingested by a *Helicobacter pylori* positive (HP+) subject, it is broken down in the stomach into ammonia and labeled CO_2_. The labeled CO_2_ is absorbed into the blood and exhaled in breathed air. Samples of the excreted CO_2_ are collected and analyzed for the presence of the isotope. Two carbon isotopes can be used: ^14^C, a radioactive isotope and ^13^C, a non-radioactive isotope.

Since its introduction by Graham et al.,^5^ and independently by Marshal and Surveyor,^6^ the UBT has been used according to a number of different protocols of varying complexity.^7,8^ In this report, we describe the evaluation of a ^14^C-UBT protocol of great simplicity, employing a simple home-made device for air collection, which therefore makes it very useful in clinical settings.

## METHODS

The study was approved by the committee for ethics in medical research of the Clinical Hospital of the Faculty of Medicine of Ribeirão Preto, University of São Paulo.

One hundred and forty-eight consecutive adult patients (age > 18 years) referred to the Clinical Hospital for upper GI endoscopy were considered for inclusion in the study, and a written informed consent was obtained from each one. Females who might have been pregnant, patients with a history of gastric surgery and those who had recently taken antibiotics or proton pump inhibitors were not included in the study. Patients with *both* the urease test and histology positive were considered HP+ and those with *both* tests negative were considered HP-. The patients with positivity in only one of these tests were excluded from the study.

### Urease test and histology

Upon endoscopy, two antral and two body mucosal biopsies were taken. The antral biopsies were taken in the greater curvature at nearly 3 cm proximal to the pylorus. One specimen from each region was used for the urease test (from the Clinical Hospital's Industrial Pharmacy) immediately after being taken. The urease test used was similar to other preparations: peptone 0.1 g; NaCl 0.5 g; glucose 0.1 g; agar 1.0 g; 2% phenol red 2.5 ml; potassium alkaline phosphate 0.2 g; urea 2 g and distilled water q.s.p. 100 ml. The remaining mucosal specimens were fixed in formalin, embedded in paraffin and stained with hematoxylin-eosin and Giemsa stain. The slides were examined by a pathologist who was unaware of the other HP test results.

## UBT

UBT was always performed within one week after endoscopy. After overnight fasting, a baseline breath sample was collected in order to ensure that the system was free of radioactivity. Immediately after this, patients ingested 185 KBq (5 μCi) of ^14^C-urea dissolved in 10 ml of water, and two additional breath samples were collected 15 and 30 minutes after ingestion. Patients blew through a plastic syringe nozzle that was, partially filled with CaCl_2_ and cotton, directly into a 20-ml glass scintillation vial containing 1.0 ml of ethanol in which 1 mmol of hyamine was dissolved and to which phenolphthalein had been added as a pH indicator. The pink (alkaline) solution became colorless upon CO_2_ saturation, at which time a constant amount (≅ 1.0 mmol) of CO_2_ had been collected.

Ten ml of scintillation solution were added to each vial and every sample was counted in a liquid scintillation counter (LS 8100 Beckman, USA). The radioactivity in each vial was expressed as counts per minute (cpm).

Using the measured cpm values and background activity from each sample, the number of disintegrations per minute (dpm) was calculated according to Marshall.^9^

### Analysis of the data

Cpm values at 15 minutes and 30 minutes after ingestion, as well as the higher of these two values for each subject in both the HP+ and HP- groups, were plotted on specific graphs and the cut-off points were determined by visual inspection of the plots. Sensitivities and specificities and their 95% confidence intervals (95% CI) were calculated using SPSS for Windows.

## RESULTS

Eleven patients were excluded from the study, as they presented with only one of the invasive tests positive, either the urease test (6 subjects) or histology (5 subjects). Out of the 137 remaining patients, 115 subjects were HP+ and 22 were HP-. The clinical and endoscopic characteristics of these groups are presented in Table 1.

**Table 1:** Clinical and endoscopic characteristics of the patients from the *Helicobacter pylori* (HP)+ and HP- groups

	HP-POSITIVE (n = 115)	HP - NEGATIVE (n = 22)
GENDER		
Female	56(48.6%)	11(50%)
Male	59(51.4%)	11(50%)
AGE (years)*	45.7±16.5 (range: 18-84)	47.8±16.7 (range: 19-76)
ENDOSCOPIC FINDINGS		
Normal	6	9
Erythematous gastritis	32	2
Erosive gastritis	32	1
Atrophic gastritis	1	1
Gastric ulcer	30	1
Duodenal ulcer	86	2
Esophagitis	18	4
Others	30	7

* *mean ± standard deviation.*

Scattergrams of cpm values for both the HP+ and HP- groups obtained 15 minutes and 30 minutes after ^14^C-urea ingestion, as well as the highest value for each subject, are given in the Figure. The figure shows that there is only a slight overlap of values between the two groups. Indeed, only 3 of the 115 HP + subjects had values lower than 1500 cpm, whereas only one HP- subject had a value higher than 1500 cpm, and in none of them was the value higher than 2000 cpm. The cpm value was higher at 30 minutes than at 15 minutes in only 9 (6.6%) subjects. Sensitivities and specificities were calculated for cut-off points of 1000, 1500 and 2000 cpm and were always higher than 90% (Table 2).

**Figure:**
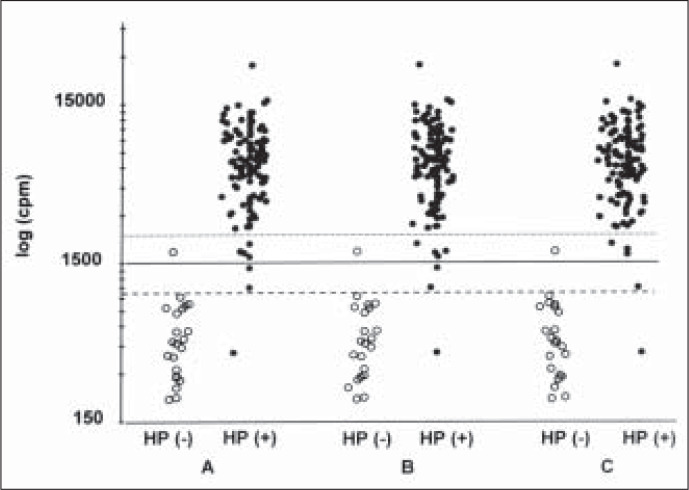
*Radioactivity in expired air of* H. pylori *positive (closed circles) and* H. pylori *negative (open circles) patients. Values are in counts per minute. A - 15 minutes after ingestion of 14 C- urea; B - 30 minutes after ingestion of 14 C- urea; C - Peak values;*

*…….. 2000 counts per minute*

*____ 1500 counts per minute*

*- - - - 1000 counts per minute.*

**Table 2:** Sensitivities and specificities of ^14^C-urea breath test for *H. pylori* using 1000, 1500 and 2000 cpm as cut-off points

	1000 cpm		1500 cpm		2000 cpm	
	sensitivity	specificity	sensitivity	specificity	sensitivity	specificity
15 minutes (95% CI)	99% (95-99)	95% (77-99)	97% (77-99)	95% (92-99)	94% (87-97)	100% (81-100)
30 minutes (95% CI)	99% (95-99)	95% (77-99)	96% (91-99)	100% (81-100)	90% (83-95)	100% (82-100)
Peak values (95% CI)	99% (95-99)	95% (77-99)	98% (93-99)	95% (77-99)	96% (90-98)	100% (82-100)

Sensitivities and specificities calculated from activities expressed as dpm were identical to those calculated from cpm.

## DISCUSSION

The results of the present study demonstrate that HP infection can be accurately detected by means of a simplified UBT. This UBT is capable of discriminating between HP+ and HP- subjects in a way similar to that of the combination of histology plus the urease test. In fact, when the combination of histology and urease test is taken as the "gold standard" for HP infection, the calculated sensitivity and specificity of UBT are higher than 90%, and often higher than 95%. These results confirm the findings of Marshall et al.,^7^ indicating the usefulness of a rapid and easy-to-perform UBT. Moreover, our results demonstrate that a very simple device for collecting the expired air can be employed with no loss of accuracy in the test.

The findings in this study indicate that the accuracy of the UBT does not depend on either a complex test meal or the calculation of total CO_2_ body production, as proposed by some authors.^8,10^ In fact, the ingestion of the urea diluted in a small volume of plain water, presumably because of making the contact of the isotope with the gastric mucosa easier, is associated with a peak of radioactivity within 30 minutes, which is thus earlier than that seen after a calorie-rich test meal. This is relevant from a practical point of view because the test is easier to perform, saving time for the patient and the technician involved.

It is noteworthy that the ^14^CO_2_ excretion was higher at 15 minutes than at 30 minutes after ^14^C-urea ingestion for more than 90% of the subjects. However, there was no significant difference between either the sensitivities or specificities calculated from the data obtained at each of these two times. This finding is consistent with the data of Marshall et al.,^7^ which showed a ^14^CO_2_ plateau from 10 minutes and up to 20 minutes after ^14^Curea ingestion.

In our laboratory, measured background activities were much lower than all activities measured in samples of expired air obtained after ^14^C-urea ingestion. As a consequence, distributions of sensitivities and specificities calculated from dpm were identical to those calculated from cpm. This finding indicates that dpm calculation is useless from a practical standpoint.

It has been proposed that enhancement of the accuracy of UBT is obtained by supplying ^14^C-urea in a capsule to avoid false-positive results from urease-producing bacteria in the oropharynx.^11^ However, oral ^14^CO_2_ production after ^14^C-urea ingestion is immediate and short-lived, lasting less than 15 minutes.^7^ Our results are consistent with these data, since overlapping between results from HP+ and HP- subjects occurred by a down-skewed distribution of values from HP + subjects, rather than by locating values of HP-within the range defined by the bulk of the numerical results obtained from the HP^+^.

Finally, on the basis of our results, either 1000 cpm or 1500 cpm should be chosen as the cut-off value, depending on whether high sensitivity or high specificity, respectively, is required.

This study was carried out using ^14^C-urea, which imposes a small but safe^12^ burden of ionizing radiation on the patients. Since it is radiation-free, ^13^C-urea has been taking the place of ^14^C-urea in breath tests for *H. pylori*. It is important to note that these two isotopes are very similar and so they produce similar results.

## CONCLUSION

The rapid UBT employing a simple device for air collection has a high accuracy in determining HP infection.
